# Concomitant Treatment with Doxycycline and Rifampicin in Balb/c Mice Infected with *Brucella abortus* 2308 Fails to Reduce Inflammation and Motor Disability

**DOI:** 10.3390/ph17050638

**Published:** 2024-05-15

**Authors:** José Luis Maldonado-García, Samantha Alvarez-Herrera, Gilberto Pérez-Sánchez, Enrique Becerril-Villanueva, Lenin Pavón, Emiliano Tesoro-Cruz, Manuel Iván Girón-Pérez, Gabriela Hurtado-Alvarado, Gabriela Damián-Morales, Rubén López-Santiago, Martha C. Moreno-Lafont

**Affiliations:** 1 Laboratorio de Inmunología Celular, Departamento de Inmunología, Escuela Nacional de Ciencias Biológicas, Instituto Politécnico Nacional, Mexico City 11340, Mexico; joselmgarci@comunidad.unam.mx (J.L.M.-G.); damianunam@hotmail.com (G.D.-M.); rlopezsantiago@hotmail.com (R.L.-S.); 2 Departamento de Bioquímica, Facultad de Medicina, Universidad Nacional Autónoma de México, Mexico City 04360, Mexico; 3 Laboratorio de Psicoinmunología, Dirección de Investigaciones en Neurociencias del Instituto Nacional de Psiquiatría Ramón de la Fuente, Mexico City 14370, Mexico; dra.alvarezherrera@gmail.com (S.A.-H.); gilberto.perez.sanchez@inprf.gob.mx (G.P.-S.); lusenbeve@inprf.gob.mx (E.B.-V.); 4 Unidad de Investigación Biomédica en Inmunología e Infectología, Hospital de Infectología, Centro Médico Nacional “La Raza”, IMSS, Mexico City 02990, Mexico; emiliano_tesoro@hotmail.com; 5 Laboratorio Nacional LANIIA-Nayarit, Universidad Autónoma de Nayarit, Tepic 63173, Mexico; ivangiron@uan.edu.mx; 6 Departamento de Anatomía, Facultad de Medicina, Universidad Nacional Autónoma de México, Mexico City 04360, Mexico; hurtadoalvg@comunidad.unam.mx

**Keywords:** brucellosis, doxycycline, rifampicin, behavioral test, inflammatory response, neurotransmitters

## Abstract

Brucellosis is an infection widely distributed around the world, and in some countries it is considered a public health problem. Brucellosis causes insidious symptoms that make it difficult to diagnose. Infection can also trigger chronic pain and neuropsychiatric complications. Antibiotics are not always effective to eradicate infection, contributing to chronicity. We aimed to investigate the effects of antibiotic treatment on proinflammatory cytokines, neurotransmitters, corticosterone, and behavior in a murine model of infecrion of *B. abortus* strain 2308. Four study groups were created: (a) control; (b) antibiotic control; (c) infected with *B. abortus* 2308; and (d) infected and treated with rifampicin and doxycycline. We determined *B. abortus* 2308 colony-forming units (CFUs), the count of dendritic cells, and macrophages in the spleen; serum levels of cytokines and corticosterone; levels of serotonin, dopamine, epinephrine, and norepinephrine in the brain; and equilibrium, physical strength, anxiety, and hopelessness tests. The infected and treated mice group was compared with the control and infected mice to assess whether treatment is sufficient to recover neuroimmunoendocrine parameters. Our results showed that despite the treatment of brucellosis with rifampicin and doxycycline, antibiotic-treated mice showed a persistence of *B. abortus* 2308 CFUs, an increased count in macrophage number, and higher circulating levels of corticosterone. Furthermore, the levels of IL-12, IL-6, and TNF-α remained higher. We found a decrease in muscular strength and equilibrium concomitant to changes in neurotransmitters in the hippocampus, cerebellum, and frontal cortex. Our data suggest that the remaining bacterial load after antibiotic administration favors inflammatory, neurochemical, and behavioral alterations, partly explaining the widespread and paradoxical symptomatology experienced by patients with chronic brucellosis.

## 1. Introduction

Brucellosis is a disease caused by the gram-negative bacteria of the genus *Brucella*. It causes chronic pain, anxiety, and depression, affecting the life quality of patients. This zoonosis has a wide geographic distribution, with over 170 countries reporting brucellosis, and 500,000 new cases annually, according to the World Health Organization (WHO) [1]. However, reported cases are only a fraction of the actual number, which is estimated to be 10–25 times higher [2,3]. Brucellosis is highly present in Latin America, Africa, Asia, and the Mediterranean region. In humans, this infection induces non-specific symptoms such as fever, headache, myalgia, arthralgia, anorexia, fatigue, weight loss, and lymphadenopathy [4]. Brucellosis has been associated with negative economic effects in countries where it is endemic due to transmission in livestock production [2,5].

The main objective of the treatment of human brucellosis is to control the disease, preventing complications and relapses. In the context of brucellosis, the goal of treatment is adequate antibiotic therapy with prolonged duration; however, the most effective antibiotic combination and duration of treatment remain unclear [6,7]. Additionally, there are limitations to choosing the best treatment scheme due to the need to choose antibiotics that act intracellularly and avoid relapses with prolonged therapy that can lead to increased adverse drug effects [8]. Antibiotic monotherapy for human brucellosis is considered inadequate due to unacceptably high relapse rates. Thus, two-antibiotic therapies are preferred [6,8], although some authors have reported that triple-antibiotic therapy could be more effective [7].

Treatment for human brucellosis is based on combinations of antibiotics such as doxycycline, streptomycin, gentamicin, rifampin, ciprofloxacin, and trimethoprim–sulfamethoxazole. An example of an antibiotic scheme to treat human brucellosis is doxycycline 100 mg orally twice daily for 6 weeks, plus streptomycin 1 g intramuscularly once daily for the first 14–21 days (or gentamicin 5 mg/kg for 5–14 days). Another combination used is doxycycline 100 mg orally twice daily, plus rifampin 600–900 mg (15 mg/kg) orally once daily for 6 weeks. For triple therapy, amikacin is added intramuscularly twice a day for 7 days in order to alleviate symptoms more rapidly [2,6,7,8,9,10,11]. Many studies have reported a significant relapse rate associated with these treatments [10]. In murine models, treatment schemes combining doxycycline and oral rifampicin are used; gentamicin and streptomycin have also been used intraperitoneally [12]. A combination of doxycycline (2 mg/day, orally) and rifampicin (0.5 mg/day, orally) for 14 days was used in this study [12].

A possible mechanism of *Brucella*’s antibiotic resistance is its permanence in intracellular compartments of reticuloendothelial cells or immune sanctuaries, such as bone marrow, which makes it difficult for most antibiotics to eradicate the bacteria [11,13].

The treatment of brucellosis may face hindrances due to the challenges in accessing antibiotics such as streptomycin in several regions across the globe, and the risk of developing resistance to rifampicin, particularly in tuberculosis-endemic areas. [9]. Moreover, the ideal duration of antibiotic therapy for bacterial elimination is yet to be defined, as prolonged treatment may lead to adverse effects and decrease therapeutic adherence [2,8]. In certain patients, *Brucella*’s genetic material was detected through qPCR analysis even years after treatment [14]. The estimated incidence of relapse has been reported to range between 5 and 30% of patients [2,15]. Most patients typically recover within a year following treatment; however, a small but notable proportion of patients may continue to experience clinical manifestations [15]. Like humans, mice infected with brucellosis respond partially to antibiotic treatment, but may develop chronic disease [16].

Brucellosis infection in mice increases anxiety and depression-like behavior. This change is associated with disturbances in the neurochemical and peripheral immunological systems. Previously, our group reported a decrease in serotonin and dopamine levels in the hippocampus and frontal cortex and an increase in peripheral cytokines such as IL-6, IL-12, IFN-γ, and TNF-α [17]. It should be noted that, in recent years, there has been increasing evidence that associates the inflammatory response with the appearance of neuropsychiatric complications in humans and behavioral changes in animal models through various mechanisms, such as a decrease in the production of serotonin or dopamine in the brain [18,19]. The aim of this study is to explore the molecular mechanisms that trigger the physical and behavioral changes responsible for the development of chronic brucellosis and its related symptoms, such as motor dysfunction. We conducted research on mice infected with *B. abortus* 2308 to examine the effects of antibiotic treatment on brucellosis, and found that even after administering antibiotics, the remaining bacterial load can cause inflammation, leading to neurochemical changes and behavioral alterations.

## 2. Results

**Figure 1:**
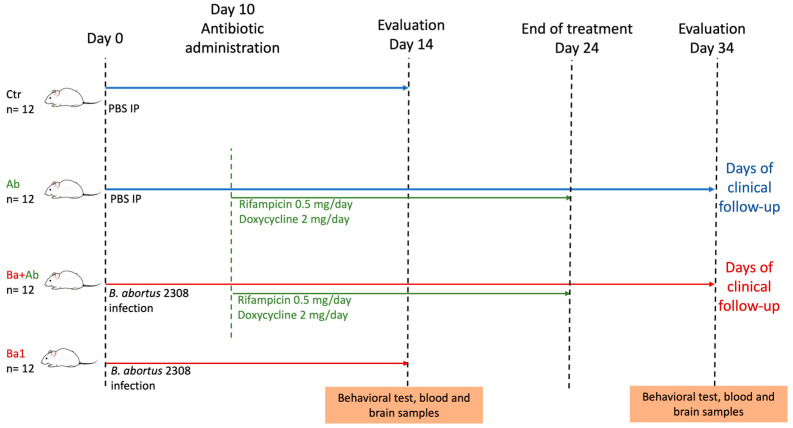
Experimental design. Blue lines indicate the follow-up time of the Ctr and Ba groups. Red lines indicate the follow-up time of the Ab and Ba + Ab groups, while green lines indicate the duration of antibiotic treatment. Ctr: Control group. Ab: Antibiotic group. Ba: Infected group. Ba + Ab: Infected and treated group.

Four groups of Balb/C mice received the following treatments: (i) a control group (Ctr group), inoculated with PBS intraperitoneally and treated only with PBS intragastrically; (ii) a group inoculated with PBS and treated with antibiotics (Ab group); (iii) a group infected with *B. abortus* 2308 intraperitoneally and treated with PBS intragastrically (Ba group); and (iv) a treated group infected with *B. abortus* 2308 intraperitoneally and treated with PBS intragastrically. The antibiotic treatment used in this work was a combination of rifampicin and doxycycline (Figure 1). The antibiotic was administered for 14 days in the Ab and Ba + Ab groups. The Ctr and Ba groups were euthanized at day 14 post-infection/inoculation. In contrast, the Ab and Ba + Ab mice were euthanized 10 days after antibiotic treatment to mitigate any potential inhibitory effects stemming from the continued presence of antibiotics in their tissues, as previously reported by Grilló et al. [12]. The purpose of comparing these study groups was to evaluate the status of the acute infection (Ba group) and the status of the infected mice who received pharmacological treatment and underwent subsequent evaluation (Ba + Ab group). This is because in human brucellosis there is a relapse rate after treatment in which symptoms such as myalgias, arthralgias, fatigue, or malaise appear [2,20].

### 2.1. Antibiotic Therapy in Mice Infected with B. abortus 2308 Does Not Restore TNF-α, IL-6, or IL-12 Levels, despite the Increase in Splenic Macrophages and Dendritic Cells

**Figure 2:**
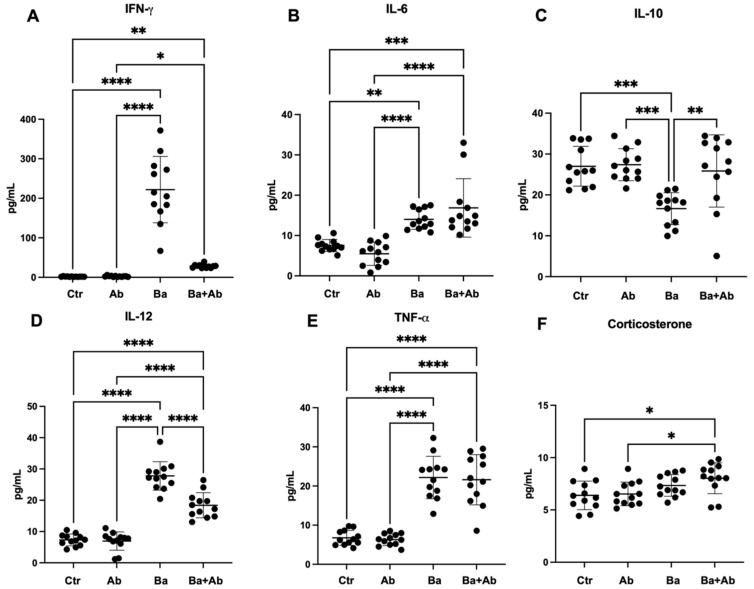
Combination treatment with doxycycline and rifampin does not decrease serum IL-6, IL-12, or TNF-α. Antibiotic treatment causes a significant decrease in IFN-γ levels in infected mice (**A**). Ba + Ab mice maintained high levels of IL-6 despite antibiotic treatment (**B**). Antibiotic treatment restored IL-10 levels in the Ba + Ab group mice (**C**). Despite antibiotic treatment to eradicate the infection, the mice in the Ba + Ab group had elevated levels of IL-12 (**D**) and TNF-α (**E**). Mice in the Ba + Ab group showed higher corticosterone levels after antibiotic treatment (**F**). Graph shows the mean ± SD of control (Ctr), antibiotic control (Ab), *B. abortus* 2308-infected (Ba), and infected and treated with antibiotics (Ba + Ab) groups. In all cases, the ANOVA test with Tukey’s post hoc test was performed, except for IL-6 and IFN-γ, where the Kruskal–Wallis test with Dunn’s post hoc test was performed. Significance is represented as follows: * *p* < 0.05, ** *p* < 0.01, *** *p* < 0.001, **** *p* < 0.0001.

The statistical analysis showed significant differences in the circulating levels of IFN-γ (H(3) = 40.07, *p* < 0.0001) between the four groups examined. As expected, the Ba mice group had an increase in IFN-γ in comparison with the Ctr group (*p* < 0.0001), and when comparing the Ba + Ab and Ba groups, it was observed that treatment with antibiotics decreased the concentration of IFN-γ (*p* < 0.0001) (Figure 2A). Statistically significant differences were observed in IL-6 concentration (H(3) = 36, *p* < 0.0001). The Ba mice group had a higher IL-6 concentration compared to the Ctr mice (*p* < 0.01), and when mice in the Ba + Ab group were compared with those in the Ba group, no statistically significant differences were observed. However, when the Ba + Ab group was compared to the Ctr group, a higher concentration of IL-6 was quantified (*p* < 0.001) (Figure 2B). Also, statistically significant differences were found in serum IL-10 determination (F = 9.4 df, n (3, 44), *p* < 0.0001). *B. abortus* 2308 infection induced a decrease in peripheral IL-10 concentration in the Ba group mice when compared to the Ctr group (*p* < 0.001). It was observed in the Ba + Ab group mice that antibiotic treatment caused an increase in serum IL-10 when compared to the Ba + Ab group mice (*p* < 0.01) (Figure 2C). Another statistically significant difference was found in the IL-12 concentrations in the groups evaluated (F = 97.59 df, n (3, 44), *p* < 0.0001). A higher concentration of IL-12 was quantified in the infected mice of the Ba group in comparison with the Ctr group (*p* < 0.001). When the Ba + Ab group was compared with the Ba group, it was observed that the antibiotic therapy induced a decrease in IL-12 concentration (*p* < 0.001), but when the values were compared with the control group, it was observed that the Ba + Ab group had a higher IL-12 concentration (*p* < 0.001) (Figure 2D). Finally, significant differences were observed in the comparison of serum levels of TNF-α (F = 49 df, n (3, 44), *p* < 0.0001). Infection induced an increase in TNF-α in the Ba group compared to the Ctr group (*p* < 0.001). No significant differences in TNF-α concentration were obtained when comparing the Ba mice to the Ba + Ab group mice; therefore, when comparing the Ba + Ab and Ctr groups, it was observed that TNF-α levels remained elevated despite treatment (*p* < 0.001) (Figure 2E). Additionally, blood corticosterone concentration was quantified. Previously, it has been reported that brucellosis does not modify serum corticosterone concentrations in humans and mice [17,21]; however, administering antibiotics significantly increased serum corticosterone levels (F = 4.4 df, n (3, 44), *p* < 0.01) in the Ba + Ab group compared to the Ctr (*p* < 0.05) and Ab (*p* < 0.05) groups (Figure 2F).

**Figure 3:**
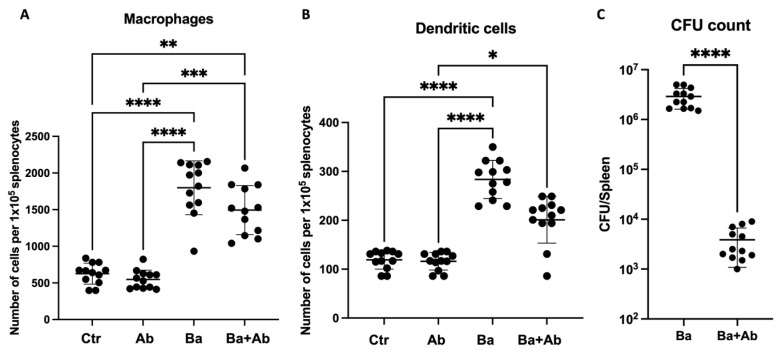
Antibiotic administration increases macrophage count but does not eliminate infection. After the administration of antibiotic treatment, it was observed that the macrophage (**A**) and dendritic cell (**B**) counts in the mice of the Ba + Ab group remained elevated, similar to the Ba group. (**C**) Antibiotic treatment significantly decreased *B. abortus* 2308 colony-forming units (CFUs) in the spleens of mice in the Ba + Ab group compared to mice in the Ba group, but did not eliminate infection. Graph shows the mean ± SD of control (Ctr), antibiotic control (Ab), *B. abortus* 2308-infected (Ba), and infected and treated with antibiotics (Ba + Ab) groups. Kruskal–Wallis test with Dunn’s post hoc test was performed in all cases, except for CFU count were Mann–Whitney U test was performed. Significance is represented as follows: * *p* < 0.05, ** *p* < 0.01, *** *p* < 0.001, **** *p* < 0.0001.

The numbers of dendritic cells and splenic macrophages, as well as CFUs in the spleen, were quantified to evaluate the immune activity and killing capacity of *B. abortus* 2308 after antibiotic treatment. The count of splenic macrophages showed a statistically significant difference (H(3) = 36.79, *p* < 0.0001). As expected, *B. abortus* 2308 infection induced an increase in splenic macrophages in Ba mice compared to Ctr mice (*p* < 0.0001), but when compared to Ba + Ab mice no difference was observed. After treatment, Ba + Ab mice had a higher macrophage count compared to Ctr mice (*p* < 0.01) (Figure 3A). Similarly, the splenic dendritic cell count showed statistically significant differences (H(3) = 33.85, *p* < 0.0001). A higher dendritic cell count was found in the spleens of the Ba group mice compared to the Ctr mice (*p* < 0.0001), while after antibiotic treatment it was observed that the Ba + Ab group mice had a lower dendritic cell count than the Ba group mice (*p* < 0.05) but a higher count than the Ctr group mice (*p* < 0.05) (Figure 3B). Concerning the *B. abortus* 2308 CFU count in the spleen, a significant difference in the CFU count between the Ba and Ba + Ab groups was found (U = 0.0, *p* < 0.0001). Despite the combined therapy of doxycycline and rifampicin for 14 days, antibiotic use did not totally eradicate *B. abortus* 2308 in the Ba + Ab group, although a significant decrease was observed (*p* < 0.0001) (Figure 3C).

### 2.2. Antibiotic Treatment of B. abortus 2308-Infected Mice Restores Neurotransmitter Levels, but Not Physical Performance

**Figure 4:**
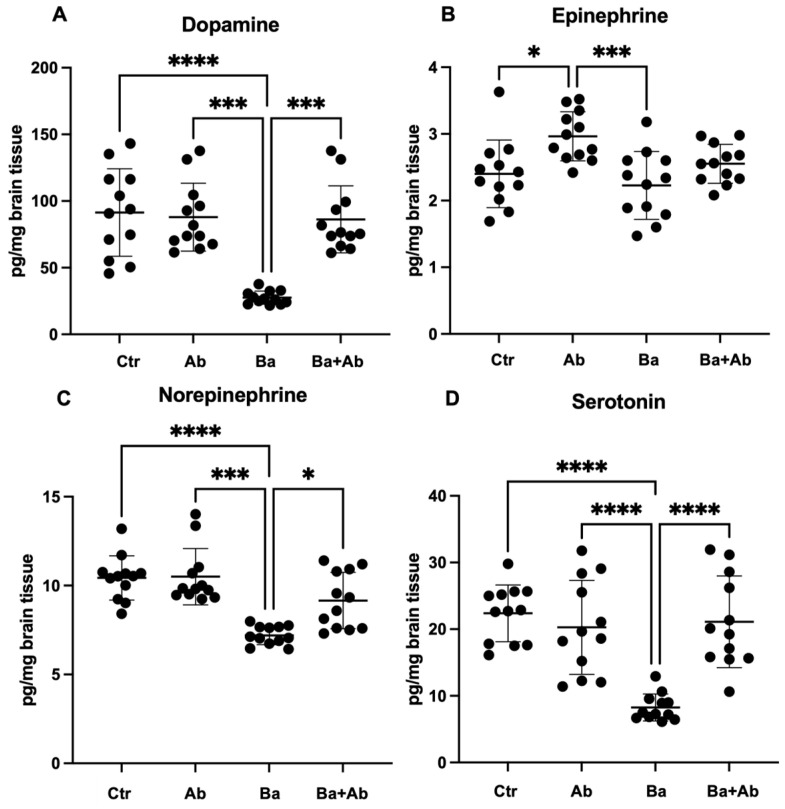
Antibiotic treatment with doxycycline and rifampicin restores hippocampal neurotransmitter levels. *Brucella* infection caused a decrease in dopamine (**A**), norepinephrine (**C**), and serotonin (**D**) levels in the Ba group mice in comparison with controls. However, after antibiotic treatment administration there was a dopamine (**A**), norepinephrine (**C**), and serotonin (**D**) increase when comparing Ba + Ab and Ba groups. Antibiotic administration induced an increase in epinephrine concentration (**B**). Graph shows the mean ± SD of control (Ctr), antibiotic control (Ab), *B. abortus* 2308-infected (Ba), and infected and treated with antibiotics (Ba + Ab) groups. In all cases, the ANOVA test with Tukey’s post hoc test was performed, except for dopamine and norepinephrine, where the Kruskal–Wallis test with Dunn’s post hoc test was performed. Significance is represented as follows: * *p* < 0.05, *** *p* < 0.001, **** *p* < 0.0001.

We found that the effect of *B. abortus* 2308 infection and antibiotic treatment on neurotransmitter levels, such as norepinephrine, epinephrine, dopamine, and serotonin, varied depending on the region of the brain. In the case of the hippocampus, significant differences were detected for norepinephrine (H(3) = 24.64, *p* < 0.0001), epinephrine (F = 17.31 df, n (3, 44) *p* < 0.0001), dopamine (H(3) = 26.54, *p* < 0.0001), and serotonin (F = 17.31 df, n (3, 44) *p* < 0.0001). The Ba group mice had lower dopamine concentrations compared to the Ctr group mice (*p* < 0.0001), while the antibiotic treatment induced an increase in dopamine in the Ba + Ab mice compared to the Ba group mice (*p* < 0.001) (Figure 4A). No differences were found in epinephrine concentrations caused by infection in the Ba group in comparison to the Ctrl group; however, it was found that mice in the Ab group had a higher concentration of epinephrine compared to the Ctr (*p* < 0.05) and Ba (*p* < 0.001) groups (Figure 4B), suggesting that drug treatment may cause an increase in hippocampal epinephrine levels. The infection caused a decrease in norepinephrine levels in the Ba group compared to the Ctr group (*p* < 0.0001), while the Ba + Ab group, after receiving antibiotic treatment, had higher norepinephrine levels than the Ba group (*p* < 0.05) (Figure 4C). In a similar way to that observed in dopamine and norepinephrine, infection caused a decrease in serotonin levels in the Ba group mice compared to the Ctr group (*p* < 0.0001), and there was a significant increase in the Ba + Ab group compared to the Ba group (*p* < 0.0001) (Figure 4D).

**Figure 5:**
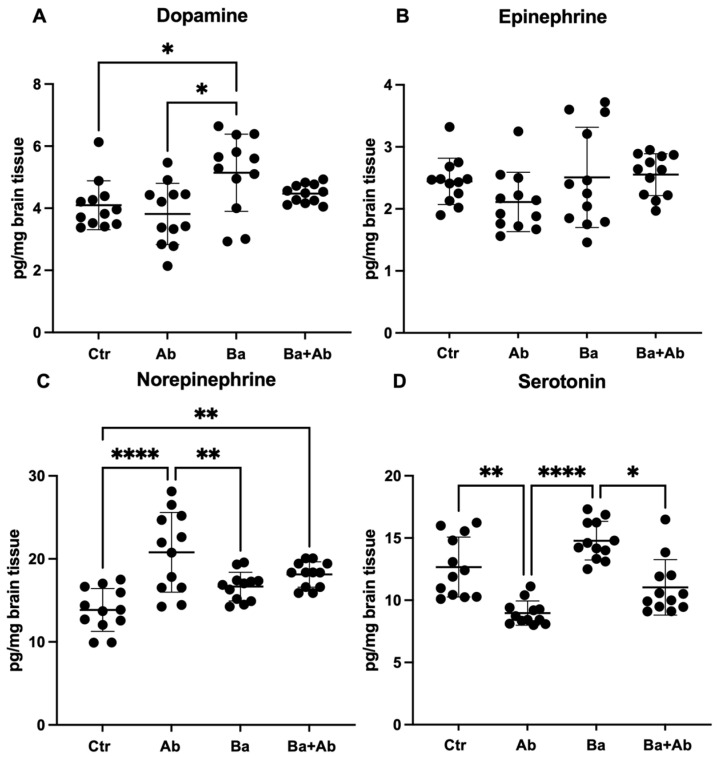
Concomitant treatment with doxycycline and rifampicin restores neurotransmitter levels in cerebellum. *Brucellosis* caused an increase in dopamine levels (**A**). Epineprine showed no changes due to infección (**B**). Results suggest that antibiotic treatment induced higher levels of norepinephrine in Ab and Ba + Ba groups (**C**). Meanwhile, Ab and Ba + Ab groups reported lower levels of serotonin (**D**). Graph shows the mean ± SD of control (Ctr), antibiotic control (Ab), *B. abortus* 2308-infected (Ba), and infected and treated with antibiotics (Ba + Ab) groups. In all cases, the ANOVA test with Tukey’s post hoc test was performed, except for dopamine and norepinephrine, where the Kruskal–Wallis test with Dunn’s post hoc test was performed. Significance is represented as follows: * *p* < 0.05, ** *p* < 0.01, **** *p* < 0.0001.

In the cerebellum, significant differences were found in the levels of dopamine (H(3) = 11.20, *p* < 0.05), norepinephrine (F = 11.47 df, n (3, 44) *p* < 0.0001), and serotonin (H(3) = 29.25, *p* < 0.0001). The infection induced an increase in dopamine concentrations in the Ba group compared with the Ctr group (*p* < 0.05) (Figure 5A). Meanwhile, epinephrine concentrations did not show statistically significant differences between the groups evaluated (Figure 5B). There was no evidence of changes in norepinephrine concentrations induced by infection. However, the concentration of norepinephrine in the cerebellum of mice in the Ab group was found to be higher compared to the Ctr (*p* < 0.0001) and Ba (*p* < 0.01) groups. Additionally, it was observed that the mice of the Ba + Ab group had higher levels of norepinephrine than the Ctr group (*p* < 0.01), so these results suggest that the administration of rifampicin and doxycycline could influence the production of norepinephrine in the cerebellum (Figure 5C). The infection did not modify serotonin levels in the Ba group compared to the Crt mice. However, the Ab group had lower serotonin levels than the Ctr (*p* < 0.01) and Ba (*p* < 0.0001) groups. Additionally, it was observed that the Ba + Ab group had lower serotonin levels than the Ab group (*p* < 0.05) (Figure 5D).

**Figure 6:**
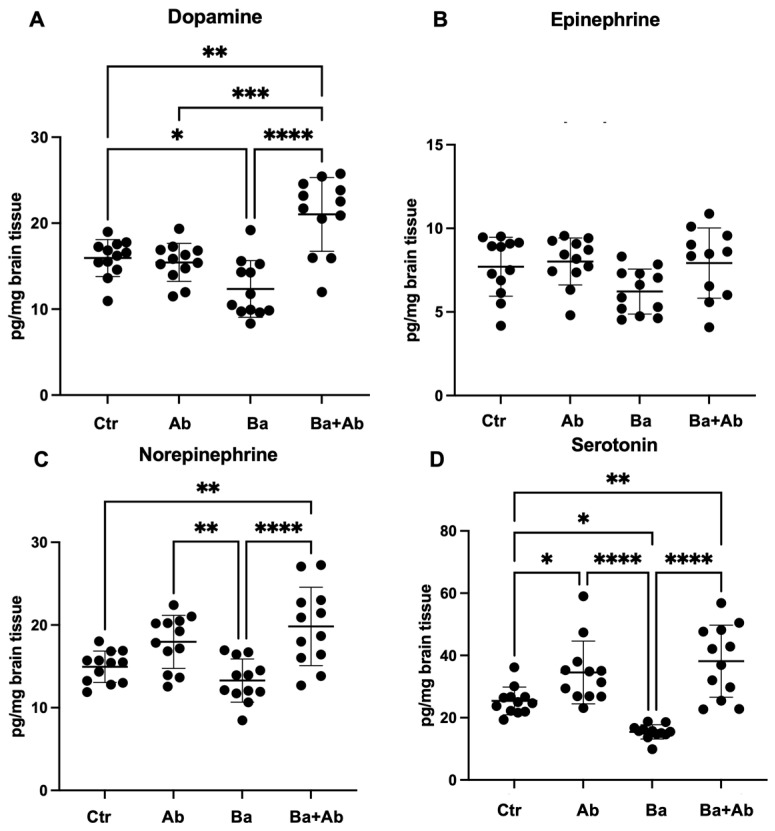
Treatment with doxycycline and rifampicin restores neurotransmitter levels in frontal cortex. *Brucella* infection caused a decrease in dopamine (**A**) and serotonin (**D**) levels in the Ba group mice in comparison with controls. Antibiotics treatment in Ba + Ab group induced higher levels of dopamine (**A**), norepinephrine (**C**), and serotonin (**D**) in comparison with Ctr and Ba groups. Epineprine showed no changes (**B**). Graph shows the mean ± SD of control (Ctr), antibiotic control (Ab), *B. abortus* 2308-infected (Ba), and infected and treated with antibiotics (Ba + Ab) groups. In all cases, the ANOVA test with Tukey’s post hoc test was performed, except for dopamine and serotonin, where the Kruskal–Wallis test with Dunn’s post hoc test was performed. Significance is represented as follows: * *p* < 0.05, ** *p* < 0.01, *** *p* < 0.001, **** *p* < 0.0001.

In the frontal cortex, we found changes in the levels of dopamine (F = 15.93 df, n (3, 44) *p* < 0.0001), norepinephrine (F = 9.64 df, n (3, 44) *p* < 0.0001), and serotonin (F = 18.97 df, n (3, 44) *p* < 0.0001). *B. abortus* 2308 infection decreased dopamine concentration in the Ba group compared to the Ctr group (*p* < 0.05). The antibiotic treatment in the Ba + Ab group increased dopamine levels (*p* < 0.0001) in comparison with the Ba group, and interestingly, the Ba + Ab mice also had higher dopamine concentrations than the Ctr mice (*p* < 0.01), suggesting that this effect of an increase in dopamine levels is present only in infected animals that receive treatment (Figure 6A). The quantification of epinephrine in the frontal cortex showed no statistically significant changes (Figure 6B). In addition, no significant differences were found for norepinephrine between the Ba and Ctr groups; however, similar to that observed for dopamine, the Ba + Ab group mice had a higher concentration of norepinephrine compared to the Ba (*p* < 0.0001) and Ctr (*p* < 0.01) groups. In addition, the Ab group had higher concentrations of norepinephrine compared to the Ba group (*p* < 0.01) (Figure 6C). Finally, serotonin levels in the frontal cortex were lower in the Ba group compared with the Ctr group (*p* < 0.01) and the Ba + Ab group, which showed higher serotonin levels (*p* < 0.0001). The Ba + Ab group had higher serotonin levels than the Ctr group (*p* < 0.01), and similarly, the Ab group had an increase in serotonin compared to the Ctr group (*p* < 0.05) (Figure 6D).

**Figure 7:**
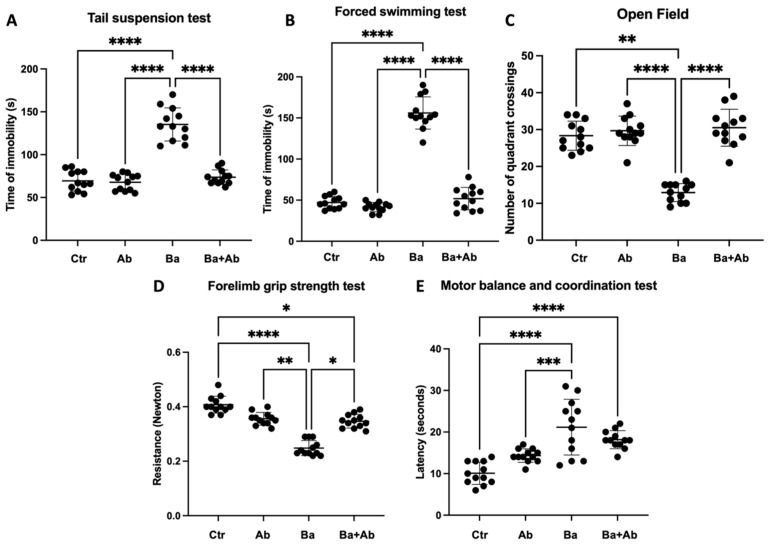
Treatment with doxycycline and rifampicin improves anxiety and hopelessness but not physical performance. Tail suspension test (**A**) and forced swimming test (**B**) evaluated hopelessness, which improved after antibiotic treatment. The open-field test (**C**) evaluated anxiety, which improved with the administration of antibiotics. The forelimb grip strength test (**D**) and balance control test (**E**) evaluated physical performance, and it was observed that antibiotic treatment did not improve the physical condition of the mice in the Ba + Ab group, causing motor disabilities. The graph shows the mean ± SD of control (Ctr), antibiotic control (Ab), animals infected with *B. abortus* 2308 (Ba), and animals infected and treated with doxycycline and rifampicin (BA + Ab). In all cases, ANOVA with Tukey’s post hoc test was used, except for FGST and OF, where the Kruskal–Wallis test with Dunn’s post hoc test was performed. Significance is represented as follows: * *p* < 0.05, ** *p* < 0.01, *** *p* < 0.001, **** *p* < 0.0001.

Modified versions of the OF, TST, and FST were applied to evaluate hopelessness and anxiety. In addition, modified versions of the FGST and MBCT were used to evaluate physical performance through muscular resistance and balance. The TST and FST evaluate hopelessness, quantifying the immobility time of the mice in each test. The TST showed significant differences (F = 74.73 df, n (3, 44) *p* < 0.0001). Brucellosis induced an increase in immobility time in Ba mice in comparison to Ctr mice (*p* < 0.0001), and the antibiotic treatment in the Ba + Ab mice was correlated with a decrease in immobility time in comparison with the Ba mice (*p* < 0.0001) (Figure 7A). Similarly, in the FST, the differences were statically significant (F = 219 df, n (3, 44) *p* < 0.0001). The Ba group had a higher immobility time compared with the Ctr mice (*p* < 0.0001), and the Ba + Ab group showed a decrease in immobility time compared with the Ba group (*p* < 0.0001) (Figure 7B). We found significant differences in the OF test evaluating anxiety for the number of quadrant crossings (H(3) = 27.37, *p* < 0.0001). The Ba group had a lower number of quadrant crossings in comparison with the Ctr group (*p* < 0.01). The Ba + Ab group showed an increase in the test in comparison with the Ba group (*p* < 0.0001) (Figure 7C). We found significant differences in the FGST, which measures muscular endurance and strength in mice (F = 73.47 df, n (3, 44) *p* < 0.0001). *B. abortus* 2308 reduced muscular strength in the Ba group compared with the Ctr mice (*p* < 0.0001); however, although mice in the Ba + Ab group had better results than mice in the Ba group (*p* < 0.0001), the results were not similar to those observed for the Ctr group mice (*p* < 0.0001) (Figure 7D). The MBCT evaluated the coordination and balance of the mice, showing significant differences in latency (F = 19.00 df, n (3, 44) *p* < 0.0001). The infected mice in the Ba group had higher latency than the Ctr group (*p* < 0.0001). Nevertheless, the Ba + Ab mice did not improve latency in comparison with the Ba group, and showed higher latency when compared to the Ctr group (*p* < 0.0001) (Figure 7E).

Taking all the results together, it was observed that antibiotic treatment with doxycycline and rifampicin does not fully eliminate *B. abortus* 2308 infection and produces an inflammatory state. The antibiotic treatment resulted in an improvement in neurotransmitter levels, which is reflected in the improvement in hopelessness and anxiety in the Ba + Ab group mice; however, no complete physical performance improvement was observed in the mice, especially in the FGST and MBCT.

## 3. Discussion

Brucellosis is a neglected infection present in over 170 countries [22]. Despite the availability of antibiotic therapy for brucellosis, its tendencies of chronicity and persistence may lead to severely debilitating and disabling conditions [23,24]. There are two categories of patients who may develop chronic brucellosis. The first group consists of those with a focal disease, such as spondylitis or arthritis. The second group includes patients who experience a decline in their overall health, with symptoms including chronic fatigue syndrome, motor disabilities, musculoskeletal pain, depression, or anxiety [14,25,26]. In this way, chronic brucellosis impacts economic activity [5].

In this study, we found that murine *B. abortus* 2308 infection increases IFN-γ, IL-6, IL-12, and TNF-α, as reported before [17]. Interestingly, in the group treated with antibiotics, the levels of IL-6, IL-12, and TNF-α remained higher, suggesting the persistence of the inflammatory response. In human brucellosis, higher TNF-α levels have been reported in patients with brucellosis [27]. Furthermore, high IL-6 levels are associated with chronic pain in chronic inflammatory diseases [28,29], as in the case of patients with chronic brucellosis [30]. Infected mice showed a decreased performance in the FGST and MBCT. The Ba + Ab mice improved in the TST, FST, and OF test with antibiotics, but still underperformed in FGST and MBCT. This may be partially explained by the persistent increase in IL-6 levels in Ba + Ab mice. IL-6 is a cytokine highly related to the development of neuropathic pain [31]. IL-6 causes pain via sensitization and hyperexcitability by phosphorylating the Nav1.7 sodium channel [32]. Hence, the decreased performance of the Ba and Ba + Ab group mice may be caused by the pain caused by the increase in peripheral IL-6 concentration. Other proinflammatory cytokines are associated with the generation of pain, i.e., TNF-α and IL-17 [33,34]. Although an increased concentration of IL-17 in serum has been reported among patients who develop chronic brucellosis [27], our study did not analyze this factor. IL-17A has been associated with chronic pain, similar to IL-6 [35], and might explain the presence of pain in acute and chronic brucellosis. Furthermore, we previously described that the administration of imipramine in a murine model of brucellosis managed to restore muscle endurance in the FGST, in addition to decreasing IL-6 [36]. In this way, it is necessary to perform a test inside a biosafety cabinet to confirm the theory of pain in brucellosis and develop protocols to study sensitization.

An interesting finding was the elevated corticosterone levels observed in the Ba + Ab group mice. This is noteworthy since previous studies have suggested that brucellosis does not affect cortisol levels in humans or corticosterone levels in mice [17,21]. Brucellosis can lower the levels of dehydroepiandrosterone, which is responsible for promoting IL-2 release. This hormone level decrease can affect T lymphocyte activation, resulting in a less efficient immune response against infection [21,37]. In this way, elevated TNF-α and corticosterone levels are indicators of hypothalamic–pituitary–adrenal (HPA) axis hyperactivity and stress [38], since TNF-α regulates HPA axis activation [39]. Corticosterone levels were not altered in the Ba group mice despite the increased peripheral TNF-α concentration. This suggests that the increase in serum corticosterone in the Ba + Ab group may be due to the combined effect of antibiotic administration and the decrease in *B. abortus* 2308 CFUs in the spleen. However, the mice in the Ba + Ab group were older at the time of the study, so further studies are needed to understand the significance of the elevation of corticosterone in this model, which may be associated with mice aging.

We also found that *B. abortus* 2308 murine infection increases macrophage and dendritic cell count in the spleen, as previously described [36]. In Ba + Ab mice, macrophage and dendritic cell count also increased, but CFUs of *B. abortus* 2308 were still found in the spleen ten days after treatment. Studies on antibiotic treatment in murine brucellosis have shown mixed results. Rifampicin and doxycycline are 100% effective in some studies, while other studies report lower effectiveness; this phenomenon is influenced by the genetic properties of the bacteria and the host [40,41,42]. According to our observations, immediately at the end of the antibiotic treatment, no CFUs of *B. abortus* 2308 were detected in the spleen. However, when we followed up after several days, we began to isolate CFUs of the bacteria in the spleen several days after the end of treatment. A similar pattern may be occurring in patients treated for brucellosis, where a relapse is observed in 5–30% of cases, usually 1–6 months after the initial infection, and relapsed patients’ bacteria maintain the same antibiotic resistance pattern as the initial infection [10,15]. It is important to emphasize that the quantification of *B. abortus* 2308 CFUs in the spleen was performed 10 days after the end of the antibiotic treatment to mitigate any potential inhibitory effects stemming from the continued presence of antibiotics in the tissue. Our results showed that spleen CFUs of *B. abortus* 2308 were obtained in the Ba + Ab group mice, generating a persistent antigenic stimulus and inducing proinflammatory cytokine release [43].

Treatment for human brucellosis involves a combination of antibiotics such as quinolones, doxycycline, rifampicin, streptomycin, and aminoglycosides, with doxycycline in combination with rifampicin or fluoroquinolones with rifampicin being the most popular antibiotic combinations suggested by the WHO [9,44]. The problem of antibiotic-resistant *Brucella* strains has been downplayed, but some authors have highlighted this issue [45]. Antimicrobial resistance is influenced by factors like the evasion mechanisms of Brucella’s immune response. This leads to a *Brucella*-Containing Vacuole (BCV), which promotes the bacterium’s replication and survival within host cells [46,47]. Genetic factors can increase susceptibility to brucellosis and promote its chronicity [42]. In addition, it has been observed that bacteria generally maintain their antibiotic resistance over time [15,44]. Furthermore, *Brucella* strains with specific antibiotic resistance have been isolated in patients with brucellosis and subsequent relapses [15]. Another factor contributing to antibiotic resistance is the intracellular environment in which *Brucella* resides, as it can reduce the effectiveness of antibiotics [9,42]. Some drugs have drawbacks like fluctuating relapse rates, an absence of drugs in some parts of the world, the risk of resistance in tuberculosis-endemic areas, and the inability to reach *Brucella*. These factors can lead to poor adherence to treatment and promote chronicity [9]. Multi-resistant *Brucella* strains have emerged in regions where brucellosis is endemic, increasing therapeutic failure rates. Examples include Malaysia, Egypt, Qatar, and China [44]. In this way, resistance to rifampin, trimethoprim with sulfamethoxazole, ampicillin, and ampicillin–sulbactam was found in 35.71%, 32.14%, 32.14%, and 28.57% of *B. abortus* 2308 isolates, respectively [48]. To reduce relapses and chronicity, current treatment strategies for brucellosis recommend prolonged therapy with antibiotics such as doxycycline and rifampin, which are effective in the acidic intracellular environment [27]. We followed the report by Grilló et al., who treated infected mice by administering rifampicin and doxycycline orally twice a day for 14 days. Rifampicin was given at 0.5 mg/day and doxycycline at 2 mg/day [12]. Even when the treatment proposed in this work was optimal for murine brucellosis, it was far from a prolonged treatment suitable for human brucellosis. Prolonged treatments are used in human brucellosis to prevent infection relapses [6,12]. Although Grillo and their collaborators reported an effective scheme to treat murine brucellosis, we obtained different results; this may be due to differences in the microorganisms or the hosts used, for example, the brucella strain [6,12,40]. A limitation of this work is that we did not use a prolonged infection scheme as used in human brucellosis; nevertheless, this model allows us to study the effect of the antigenic stimulus secondary to the deficient elimination of the infection.

Our data also highlight the influence of antibiotic treatment on behavior and the neurotransmitter levels in the brain. Previous studies have shown that infection with *B. abortus* 2308 in mice can alter the serotonin and dopamine levels in the hippocampus and frontal cortex [36]. Our results showed that antibiotic treatment restored dopamine and serotonin levels in the hippocampus, which could result from the decrease in the antigenic stimulus caused by the decrease in *B. abortus* 2308 splenic CFUs. During the chronic phases of murine brucellosis, neurotransmitter levels in the hippocampus and frontal cortex were restored, similar to the findings previously published by our group [17]. This study raises a crucial question: whether the increase in neurotransmitter levels in the brain results from a compensatory response due to enhanced neurotransmitter production, or is due to the decrease in CFUs of *B. abortus* 2308 due to the effect of the treatment. The study observed the persistence of proinflammatory cytokines, which are known to reduce serotonin production due to the inflammatory response. Proinflammatory cytokines alter the metabolism of tryptophan, which is a precursor for serotonin synthesis in the periphery and brain [18]. Additionally, inflammation triggers oxidative stress that decreases the synthesis of tetrahydrobiopterin, which is a key cofactor to synthesize +serotonin, dopamine, and norepinephrine [18]. This leads to lower serotonin levels and an increase in kynurenine metabolites, which are associated with the development of anxiety and depression-like behavior. Our data show that the infection decreased serotonin and dopamine in the hippocampus in Ba mice, explaining the hopelessness behavior evaluated in the TST and FST [49,50]. On the other hand, Ba mice showed lower spontaneous mobility in the OF test, suggesting the development of anxiety, which can be explained by the decrease in serotonin and increase in kynurenine metabolites [51]; however, in this work, the quantification of kynurenine metabolites was not performed. When Ba + Ab mice were evaluated, there was an increase in serotonin and dopamine concentration in the hippocampus after antibiotic treatment. This may explain the decrease in hopelessness observed in the FST and TST [50]. It is suggested that increased levels of dopamine and serotonin are associated with an improvement in the infection; however, this raises new questions about the compensatory mechanisms of dopamine and serotonin production due to the persistence of elevated levels of proinflammatory cytokines in peripheral blood.

Our findings indicate that administering rifampicin and doxycycline to the mice in the Ab and Ba + Ab groups led to an increase in norepinephrine levels and a decrease in serotonin levels in the cerebellum. At the same time, the antibiotics caused an increase in the levels of dopamine, norepinephrine, and serotonin in the Ab and Ba + Ab groups. Based on our current understanding, there are no reports on the effect of rifampicin or doxycycline on neurotransmitter production in the cerebellum, making these results interesting. Taking these results with caution is important, as the quantification of neurotransmitters was performed ten days after the antibiotic scheme was completed, so further studies are needed to interpret the results. Other inflammatory stimuli, such as LPS, can induce changes in neurotransmitter production that are maintained over time [52], and there are reports of the psychiatric condition of antibiomania developing after antibiotics are consumed [53]. Antibiomania is not completely understood, but it has been proposed that it is caused by drugs stimulating areas of the brain that cause changes in neurotransmitter levels [53].

**Figure 8:**
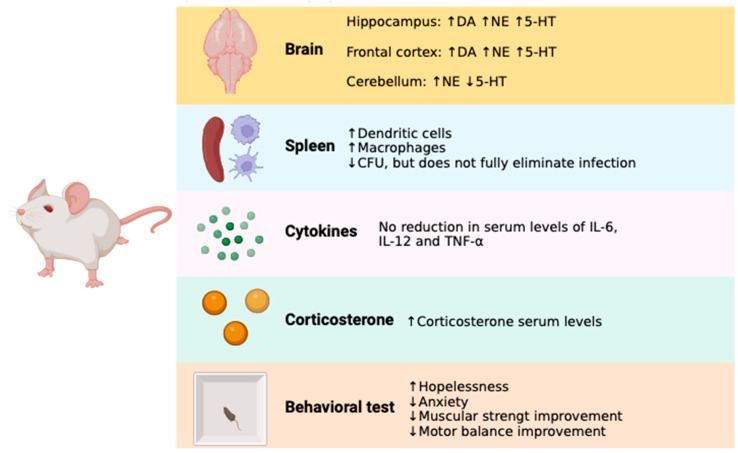
Effect of rifampicin and doxycycline administration on *B. abortus* 2308-infected mice. Upward arrows indicate improvement or increase, while downward arrows indicate worsening or decrease. CFU: colony forming unit, DA: dopamine, NE: norepinephrine, 5-HT: serotonin.

Our study found that administering antibiotics to *Brucella*-infected mice did not entirely eliminate the infection. *B. abortus* 2308 infection in our murine model decreased dopamine and serotonin in the frontal cortex and the hippocampus. In mice that underwent antibiotic treatment, we observed an increase in dopamine and serotonin levels in the hippocampus and frontal cortex, probably due to partial recovery from infection; this increase in neurotransmitter concentration manifests in a decrease in hopelessness and anxiety. However, despite the improvement in the neurotransmitter levels, the treatment did not restore muscular strength or equilibrium (Figure 8). Our model showed decreased physical performance, as evaluated by the MBCT and FGST [36], similar to chronic human brucellosis [20]. There could be various reasons for a decrease in physical performance, for instance, the persistence of high levels of IL-6 and TNF-α in the Ba + Ab group, since these cytokines are often associated with symptoms such as fatigue and pain [31,54,55]. *B. abortus* 2308 infection in mice reduces muscle endurance. Previously, our group reported that imipramine administration partially restores brucellosis-induced physical and mood disturbances [36]. Imipramine is a tricyclic antidepressant that increases serotonin concentration in the brain and decreases leukocyte IL-6 production [56]. In this model, an improvement in the physical performance of the mice was observed despite the presence of Brucella CFU in the spleen; additionally, a lower concentration of IL-6 was reported [36]. The results are interesting, as they suggest that there may be a connection between reduced serotonin levels and the occurrence of psychiatric symptoms, fatigue, and pain. Regarding this point, it has been published that, in patients with post-COVID syndrome, there is a relationship between low serotonin levels in the periphery and the development of pain, fatigue, and psychiatric complications [57]. Some authors have suggested that brucellosis could be a model for the study of post-COVID syndrome [58] since post-COVID syndrome and brucellosis share some similarities. Both conditions exhibit persistent antigenic stimuli and chronic inflammatory responses [14,59]. In this way, further studies on the psychoimmune effects of chronic infections can lead to improved patient quality of life.

To make the most of the study results, it is important to note the limitations of the research model. For example, some behavioral studies could not be performed in the biosafety cabinet, and it is necessary to implement cognitive and pain evaluations. As previously discussed, joint pain or myalgias could explain the decreased physical performance in mice as well, so future research should evaluate the effect of the infection on pain pathways to see if pain is responsible for the decline in physical performance in mice. More tests should be conducted to assess whether the changes seen in the neurotransmitter levels of the Ba + Ab mice group have any clinical relevance. The absence of follow-up research on the kinetics of the neurotransmitter changes is a significant limitation, but it presents an opportunity to further investigate antibiotic effects on the brain.

Further studies are needed to assess treatment schedules similar to those used in human brucellosis to accurately understand the mechanisms that lead to therapeutic failure and the development of comorbidities caused by neuroimmunoendocrine dysregulation. Despite the limitations of this study, it is important to note that acute or chronic infections have the capacity to induce psychiatric complications, and brucellosis is no exception [18]. For this reason, it is important to investigate pathophysiological mechanisms, especially in infections that tend to become chronic or in which pharmacological treatments do not totally eradicate the infection.

## 4. Materials and Methods

### 4.1. Brucella Culture

The strain used in this study was kindly donated by the Center for Molecular Medicine and Infectious Diseases at Virginia Polytechnic Institute and State University. Following the indication of the previous report, *B. abortus* 2308 was cultured and then quantified using the agar plate method; subsequently, a suspension of 1 × 10^6^ CFUs (colony-forming units) was obtained [17] and adjusted by optical density using a Spectronic 20 spectrophotometer (Bausch & Lomb, Laval, QC, Canada) (absorbance of 0.4 ± 0.02 at 540 nm). All procedures were performed in a Nuaire Class II type A/B3 biosafety cabinet.

#### 4.1.1. Animals

This study used 48 female BALB/c mice between 6 and 8 weeks old, weighing 18 to 20 g (supplied by Ferandelh, Mexico City, Mexico). The mice were housed in cages with three mice per group and allowed 21 days to acclimate at the animal facility of the Immunology Department at the Escuela Nacional de Ciencias Biológicas, Instituto Politécnico Nacional. The mice were allocated randomly to one of four groups: control mice without treatment (Ctr, n =12); control mice with doxycycline and rifampicin treatment (Antibiotic, Ab, n = 12); mice infected with *B. abortus* 2308 (Ba, n =12); and mice infected with *B. abortus* 2308 and treated with doxycycline and rifampicin (Ba + Ab, n =12). The Ba and Ba + Ab groups were inoculated intraperitoneally with a suspension of 1 × 10^6^ CFUs of *B. abortus* 2308 in 100 μL of 0.1 M PBS. Ctr and Ab mice groups received 100 μL of 0.1 M PBS intraperitoneally. All animals were handled in a biosafety cabinet and euthanized by cervical dislocation. The Ctr and Ba groups were euthanized on day 14. In contrast, the Ab and Ba + Ab groups were euthanized ten days after the completion of antibiotic treatment, on day 34, to avoid the inhibitory effects associated with the persistence of antibiotics in tissue [12]. The treatment scheme and sacrifice time can be referred to in Figure 1. All protocols, including mouse handling, inoculation, infection, and experimental procedures, were revised and approved by the local Animal Rights Committee (Permit No. ZOO-009-2018) and fully complied with the NIH Guide for the Care and Use of Laboratory Animals.

#### 4.1.2. Antibiotic Treatment with Doxycycline and Rifampicin

The Ab and Ba + Ab groups of mice were given doxycycline (Vibramicina, Pfizer, Mexico City, Mexico) at a dose of 2 mg/day and rifampicin (Rifadin, Sanofi Aventis, Mexico City, Mexico) at a dose of 0.5 mg/day, both diluted in PBS and administered intragastrically in accordance with Grillo et al. [12]. A stock solution was prepared in 1 mL of PBS, and the dose for each animal was calculated accordingly. The aliquot of the stock solutions was diluted with PBS to a final volume of 100 μL. The antibiotic therapy regimen began on day ten post-infection for the Ab and Ba + Ab groups and was administered daily for 14 days using an orogastric tube (Instech, Plymouth Meeting, PA, USA; Cat: FTP-20-38).

### 4.2. Collection of Blood, Brain, and Spleen Samples

On the day of completion of clinical follow-up, blood samples were collected from the facial veins of the mice into a microcentrifuge tube (Eppendorf, Enfield, CT, USA; Cat: 0030123611). Subsequently, the samples were centrifuged (2000× *g* 10 min) at room temperature. The serum was then collected, aliquoted (100 μL), and stored at −80 °C until use. Mice were subjected to cervical dislocation before decapitation. As previously described, the brain was removed from the skull, and we dissected the hippocampus, cerebellum, and frontal cortex [36]. Briefly, the skull was cut with scissors to expose the brain, and the cerebellum and olfactory bulbs were removed. The cerebellum and frontal cortex were separated using a scalpel; subsequently, the brain was placed in a ventral position to remove the thalamus and midbrain and to expose the hippocampus and separate it from the cortex. Finally, the spleen was removed for dendritic cell and macrophage determination by flow cytometry analysis and the determination of *Brucella* CFUs.

### 4.3. Serum Cytokine Quantification

A mouse inflammation kit, BD™ (catalog # 552364), was used to measure IL-6, IL-12, TNF-α, IFN-γ, and IL-10 levels, using serum samples to evaluate cytokines, as previously reported by our group [17,36]. The samples were processed using a FACSARIA III flow cytometer (BD Bioscience, San Jose, CA, USA) as per the manufacturer’s instructions. Each analysis was completed in duplicate, and the mean of the two measurements was reported.

### 4.4. Corticosterone Quantification

The level of corticosterone in the serum was measured using the corticosterone competitive ELISA Kit (Invitrogen, Waltham, MA, USA; cat# EIACORT) and evaluated through the Multiskan Go spectrophotometer (Thermo Scientific, Waltham, MA, USA). The data analysis was conducted using Skanlt, version 5.0 software (Thermo Scientific, Waltham, MA, USA).

### 4.5. Dendritic Cell and Macrophage Flow Cytometry Analysis

The spleen cell suspension was treated with ammonium chloride potassium (ACK) buffer to remove the erythrocytes. The resulting cell suspension was adjusted to 1 × 10^6^ cells and then incubated with PE anti-mouse MHC II(I-A/I-E), negative lineage (PerCP-Cy5.5 anti-mouse CD19, CD3, and B220) (BioLengend, San Diego, CA, USA), PE-CF594 anti-mouse CD11c, and V500 anti-mouse CD11b (BD Bioscience, San Jose, CA, USA) for 20 min. The cells were then washed with PBS and analyzed using a FACS-Aria III cytometer (BD Bioscience, San Jose, CA, USA) on the same day with a collection of one million events. Each fluorochrome had its own autofluorescence and compensation controls to ensure accuracy. Detritus and double events were eliminated by comparing area to size forward scatter, while granulocytic cells were selected through a plot of side scatter versus forward scatter. CD11c+, I-A/I-E+ double-positive cells were selected from this region, with CD11b-positive macrophages and Lin-, MHC-II+, and CD 11c+ dendritic cells.

### 4.6. Determination of Spleen Brucella Colony-Forming Units (CFUs)

The spleen was mechanically minced in a Petri dish with 5 mL of PBS (1 M) and meshed with a cell strainer (Cat: 431750; Corning, Corning, NY, USA). The cell suspension obtained was washed in PBS (3 mL), centrifuged, and re-suspended at room temperature. Then, 20 microliters of this cell suspension was diluted (1:10) in 1 M PBS to obtain a final dilution of 10^−4^. The samples were plated in duplicate on tryptic soy agar (TSA) and incubated for 48 h at 37 °C. Finally, the number of *Brucella* CFUs was estimated by multiplying the number of colonies by the inverse of the dilution factor.

### 4.7. Behavioral Test Evaluation

Mice from all groups underwent four behavioral tests to evaluate their neurological function and mood. The first test was a modified forelimb grip strength test (FGST), which involved measuring the strength force of tail-pulled mice while they gripped to a bar attached to a dynamometer (Labessa, Mexico City, Mexico). The mice were held in this position until they freed themselves. For the second test, mice were subjected to a modified version of the motor balance and coordination test (MBCT). They were placed on a 50 cm long and 2 cm wide bar, 30 cm above the floor, and the time spent traversing the bar was recorded (i.e., latency). The third test was the tail suspension test (TST), in which mice were suspended by their tails for 5 min, and their immobilization time was recorded when they stopped making paw movements. This test was used to assess motivation. Finally, a modified version of the open-field (OF) test was performed using a 30 cm acrylic box with nine quadrants on its floor. The mice were placed in the middle, and their exploratory behavior was recorded for 5 min. Each mouse’s number of movements across quadrants was recorded. Full quadrant occupancy was registered when the mouse placed four paws within the limits of the quadrant. The FGST and MBCT were primarily used to assess neurological conditions, while the TST and OF test were used to assess motivation.

### 4.8. Neurotransmitter Quantification

Norepinephrine, epinephrine, dopamine, and serotonin were extracted from three brain regions: the hippocampus, cerebellum, and frontal cortex. A 400 mL extraction buffer containing 5% ascorbic acid, 200 mM sodium phosphate, 2.5 mM L-cysteine, and 2.5 mM EDTA was used for this purpose. The protein was then precipitated by adding 100 uL of 0.4 M perchloric acid, followed by incubation at 20 °C for 20 min. After centrifugation at 12,419× *g* for 10 min at 4 °C, supernatants containing norepinephrine, epinephrine, dopamine, and serotonin were collected. The concentrations of NE, E, D, and serotonin were determined using reversed-phase HPLC (RP-HPLC) in a system consisting of a PU-2089 plus pump (Jasco, Inc., Easton, MD, USA), an AS-2057 plus autosampler (Jasco, Inc.), and an X-LCTM3120FP fluorescence detector (Jasco, Inc.). The instruments were controlled using ChromNav (Jasco, Inc.). Chromatographic runs were performed using a Jupiter C18 column (300 Å, 5 μm, 4.6 × 250 mm, Phenomenex, Torrance, CA, USA) at 30 °C. The column was equilibrated with mobile phase A, containing 0.1% trifluoracetic acid in water. Then, a linear gradient from minute 5 to minute 20 with mobile phase B, containing 0.1% trifluoroacetic acid in acetonitrile, was performed. The flow rate was 0.8 mL/min. The fluorescence detector was set at gain 1000, attenuation 32, response 20 s, and 280 nm and 315 nm for excitation and emission, respectively. The sample injection volume was 100 μL.

### 4.9. Statistical Analysis

The data collected from the four groups was tested for normality using the Shapiro–Wilk normality test. If the data had a Gaussian distribution, ANOVA followed by Tukey’s post hoc test was used to establish significance when *p* < 0.05. For data that had a non-Gaussian distribution, the Kruskal–Wallis test followed by Dunn’s post hoc test was used to establish significance. All statistical analysis experiments were carried out using GraphPad Prism, version 10.0.2 for MacOS (GraphPad Software, San Diego, CA, USA). The Shapiro–Wilk normality test was applied to all data collected from the four groups. Based on the results of the normality test, ANOVA followed by Tukey’s post hoc test was applied in all cases where the values had a Gaussian distribution, and the Kruskal–Wallis test followed by Dunn’s post hoc test was applied for data that had a non-Gaussian distribution. When *p* < 0.05, significance was established. GraphPad Prism, version 10.0.2 for MacOS (GraphPad Software, San Diego, CA, USA), was used for all statistical analysis experiments.

## 5. Conclusions

The treatment of brucellosis poses a significant challenge due to the multifaceted nature of the disease. Genetic factors, bacterial–host interactions, and antibiotic regimens are among the key considerations that must be considered when addressing the condition. The present study has revealed that current treatments do not entirely eradicate the infection. Despite the observed improvement in depressive symptoms in the study’s mouse model, physical sequelae were still evident. Decreased physical performance and elevated circulation levels of cytokines, such as IL-6 and TNF-α, were noted, which could exacerbate the symptomatology. Our findings suggest that the residual bacterial load following antibiotic treatment leads to inflammatory, hormonal, and behavioral changes that could partially account for the diverse and paradoxical symptomatology in patients with chronic brucellosis. As such, future research is necessary to better understand the chronic nature of the disease and develop therapeutic interventions that can improve the quality of life of affected individuals.

## Data Availability

All data are contained within the article. For any questions, please contact the authors.
